# Clinical implication of oncogenic somatic mutations in early-stage cervical cancer with radical hysterectomy

**DOI:** 10.1038/s41598-020-72518-1

**Published:** 2020-10-30

**Authors:** Takafumi Watanabe, Hideaki Nanamiya, Manabu Kojima, Shinji Nomura, Shigenori Furukawa, Shu Soeda, Daisuke Tanaka, Takao Isogai, Jun-ichi Imai, Shinya Watanabe, Keiya Fujimori

**Affiliations:** 1 Department of Obstetrics and Gynecology, Fukushima Medical University School of Medicine, Fukushima, 960-1295 Japan; 2 Medical-Industrial Translational Research Center, Fukushima Medical University School of Medicine, Fukushima, 960-1295 Japan

**Keywords:** Cancer, Genetics

## Abstract

It is well known that tumour initiation and progression are primarily an accumulation of genetic mutations. The mutation status of a tumour may predict prognosis and enable better selection of targeted therapies. In the current study, we analysed a total of 55 surgical tumours from stage IB-IIB cervical cancer (CC) patients who had undergone radical hysterectomy including pelvic lymphadenectomy, using a cancer panel covering 50 highly mutated tumorigenesis-related genes. In 35 patients (63.6%), a total 52 mutations were detected (58.3% in squamous cell carcinoma, 73.7% in adenocarcinoma), mostly in *PIK3CA* (34.5%) and *KRAS* and *TP53* (9.1%). Being mutation-positive was significantly correlated with pelvic lymph node (PLN) metastasis (*P* = 0.035) and tended to have a worse overall survival (*P* = 0.076). In particular, in the patients with squamous cell carcinoma, there was a significant association between being mutation-positive and relapse-free survival (*P* = 0.041). The patients with PLN metastasis had a significantly worse overall survival than those without (*P* = 0.006). These results indicate that somatic mutation status is a predictive biomarker for PLN metastasis in early-stage CC, and is consequently related to poor prognosis. Therefore, comprehensive genetic mutations, rather than a single genetic mutation, should be examined widely in order to identify novel genetic indicators with clinical usefulness.

## Introduction

Cervical cancer (CC) is the fourth most common cancer in women, with an estimated 570,000 new cases in 2018, representing 6.6% of all female cancers that year^1^. CC is also the fourth leading cause of cancer death in females around the world, accounting for 7.5% (315,000) of all cancer deaths among females in 2018^1^. The primary treatments for early-stage (stages I and II) CC include surgery or radiotherapy. The standard surgery for early-stage CC, except for stage IA, is radical hysterectomy (RH) with pelvic lymph node (PLN) dissection^2^. Intermediate-risk factors, including lymphovascular space invasion (LVSI), large tumour size and deep stromal invasion, do not significantly increase the recurrence rate alone; however, the presence of multiple intermediate-risk factors increases the recurrence rate to 15–20%^3,4^. Furthermore, high-risk factors such as PLN metastasis, positive surgical margins and/or parametrial invasion increase the recurrence rate to as high as 40% in postoperative CC^5^. Adjuvant treatment after RH is usually recommended to improve survival in early-stage CC patients who have pathologic risk factors^6^.

Previous studies in CC have elucidated either isolated somatic mutations or copy number alterations for several cancer-related genes, such as *PIK3CA*, *PTEN*, *TP53*, *STK11* and *KRAS*, and explored their clinical relevance^7–11^. In addition, for comprehensive elucidation of genetic alterations in cervical tumours, recurrent somatic mutations of *MAPK1*, *HLA-B*, *EP300*, *FBXW7*, *NFE2L2*, and *ERBB2* in squamous cell carcinoma (SCC), as well as those of *ELF3* and *CBFB* genes in adenocarcinoma (AC), have been identified using whole-exome sequencing analysis^12^. Some studies have reported that CC patients with mutant *KRAS* and *PIK3CA* have a poorer survival rate than those with wild-type^9,11,13,14^.

Although several studies have reported associations between somatic mutations and the clinicopathological characteristics of all CC stages , there have been few studies focusing on early-stage CC^13,15,16^. In the present study, we performed a systematic somatic mutation analysis in CC tumours using a cancer panel, and examined whether somatic mutations are associated with risk factors and prognosis in early-stage CC after RH.

## Results

### Spectrum and frequency of mutations in CC

**Figure 1:**
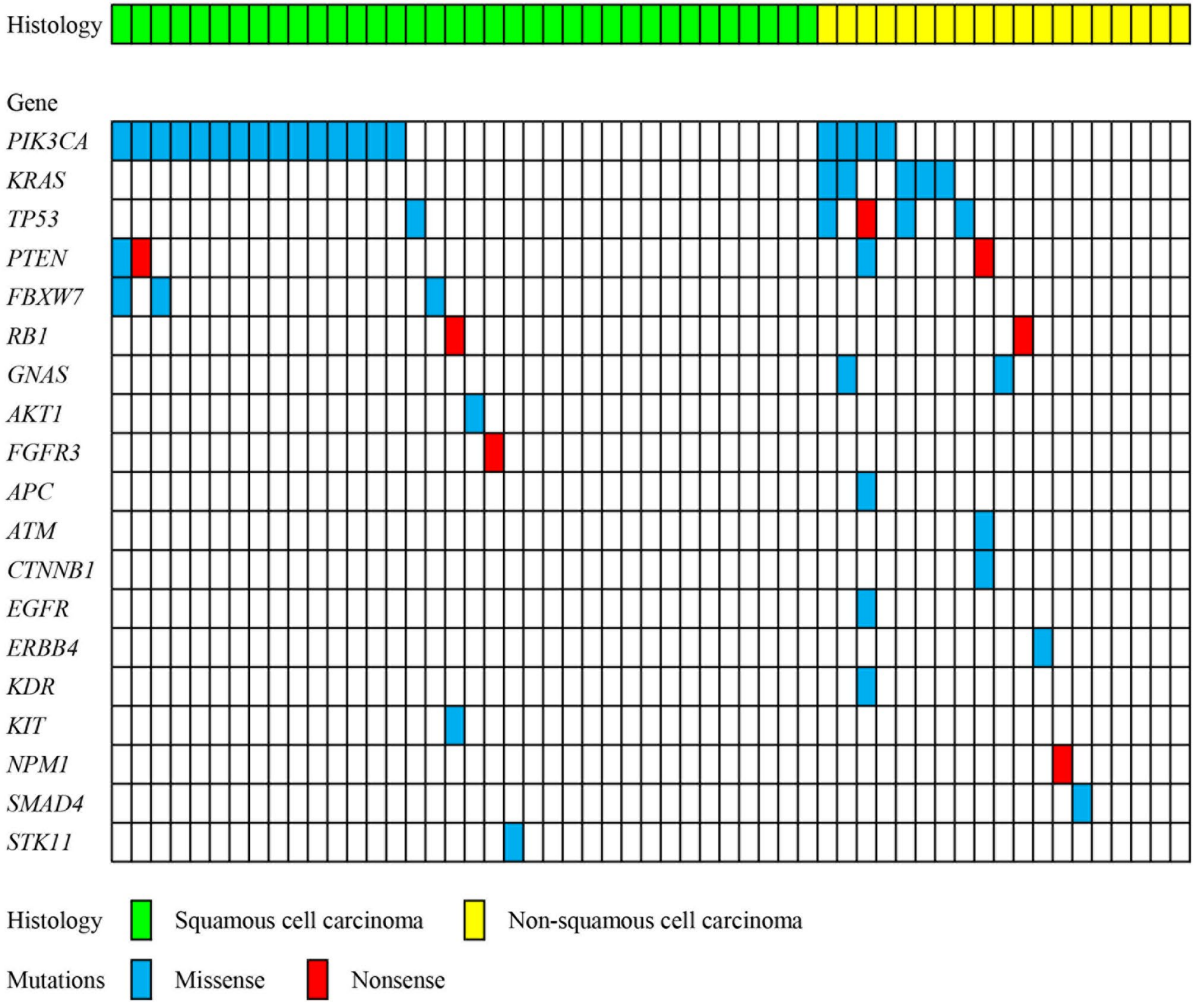
Summary of the relationships between somatic mutations and histological characteristics in cervical cancer. All panels are aligned with vertical tracks representing 55 individuals.

In order to examine the mutations of spectrum and frequency in CC, 55 frozen tumours were analysed. Figure 1 shows a summary of somatic altered genes that are recurrently mutated. Thirty-five tumours (63.6%) had at least one validated mutation, and nine (16.4%) harboured concurrent mutations in two or more genes. A total of 52 mutations were observed; missense mutations in 45 genes (86.5%) and nonsense mutations in seven genes (13.5%). The mutations per tumour were 1.37 in the non-SCC patients (AC, adenosquamous carcinoma and clear cell carcinoma, n = 19) and 0.72 in the SCC patients (n = 36). The 52 mutations were found in the following genes; *PIK3CA* (n = 19, 34.5% of the 55 tumours), *KRAS* and *TP53* (n = 5 each, 9.1%), *PTEN* (n = 4, 7.3%), *FBXW7* (n = 3, 5.5%), *RB1* and *GNAS* (n = 2 each, 2.2%), as well as in *AKT1*, *FGFR3*, *APC*, *ATM*, *CTCNB1*, *FGFR*, *ERBB4*, *KDR*, *KIT*, *NPM1*, *SMAD4* and *STK11* (n = 1 each, 1.8%).

The frequencies of somatic mutations detected in the SCC and non-SCC tumours are shown in Table 1. Of the 36 SCC patients, 21 were mutation-positive (M+) and 15 were mutation-negative (M−). Of the 19 non-SCC patients, 14 were M+ and 5 were M−. The detection rate of *PIK3CA* mutations was higher in the SCC patients compared with the non-SCC patients, although these findings were not significantly different (41.7% vs 21.1%, P = 0.126). *FBXW7* mutations were found in the SCC patients but not in the non-SCC patients (8.3% vs 0%; *P* = 0.272), although the difference was not statistically significant due to the small number of cases with M+. In contrast, *KRAS* mutations were only detected in the non-SCC patients (26.3% vs 0%; *P* = 0.003). The number of *TP53* mutations detected was also significantly higher in the non-SCC than in the SCC patients (21.1% vs 2.8%, *P* = 0.043).

**Table 1:** Frequency of mutations and histological distribution in cervical cancer.

	All n = 55	SCC n = 36	Non-SCC n = 19	*P* value
Any mutation n (%)	35 (63.6)	21 (58.3)	14 (73.7)	0.26
*PIK3CA* n (%)	19 (34.5)	15 (41.7)	4 (21.1)	0.126
*KRAS* n (%)	5 (9.1)	0 (0)	5 (26.3)	0.003
*TP53* n (%)	5 (9.1)	1 (2.8)	4 (21.1)	0.043
*PTEN* n (%)	4 (7.3)	2 (5.6)	2 (10.5)	0.43
*FBXW7* n (%)	3 (5.5)	3 (8.3)	0 (0)	0.272

### Relationship between mutation status and clinicopathological characteristics in CC

A total of 55 patients with stage IB-IIB who had been treated with RH, including pelvic lymphadenectomy, were enrolled in this study. The median age at diagnosis was 48.8 years (range 30–70 years), and 48 (87.2%) of the patients with high recurrence risk underwent post-operative radiotherapy, chemoradiotherapy or chemotherapy. Table 2 shows a summary of the associations between mutation status and clinicopathological characteristics in CC patients. The correlation between M+ and PLN metastasis was statistically significant (*P* = 0.035). However, M+ did not correlate with other clinicopathological features, such as age, tumour size, deep stromal invasion, parametrial involvement, and LVSI.

**Table 2:** Association between mutation frequencies and clinicopathological parameters of cervical cancer.

Characteristic	Total (n = 55)	Mutation positive (n = 35, 63.6%)	Mutation negative (n = 20, 36.4%)	*P* value
Age (years)				0.101
< 45	25	13	12	
≥ 45	30	22	8	
Histology				0.260
SCC	36	21	15	
Non-SCC	19	14	5	
Tumor size (mm)				0.384
< 40	18	10	8	
≥ 40	37	25	12	
Deep stromal invasion				0.168
≤ 1/2	29	16	13	
> 1/2	26	19	7	
Parametrial involvement				0.358
No	40	24	16	
Yes	15	11	4	
LVSI				0.559
No	14	8	6	
Yes	41	27	14	
Positive pelvic node				0.035
No	31	16	15	
Yes	24	19	5	

### Prognostic role of genomic mutation in CC

**Figure 2:**
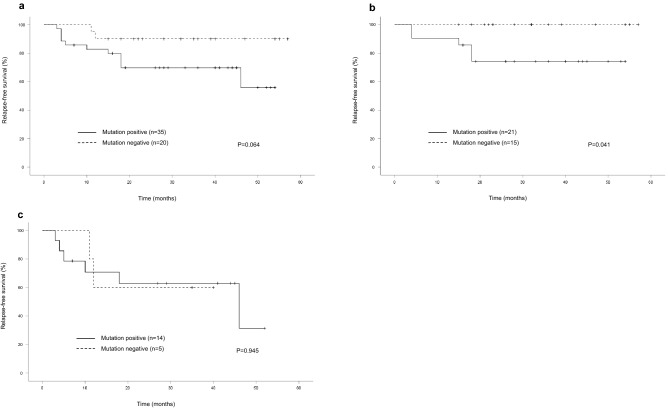
Kaplan–Meier curves showing relapse-free survival according to mutation status in patients with cervical cancer (**A**), squamous cell cervical cancer (**B**) and non-squamous cell cervical cancer (**C**).

A summary of the relationship between risk factor and survival is described in Table 3. The median follow-up period was 35 months, with a range of 7–58 months, during which 13 patients (23.6%) experienced recurrence and five patients (9.1%) died. The 3-year relapse-free survival (RFS) and overall survival (OS) were 78.2% and 90.9%, respectively. Histology, parametrial involvement, and PLN were significantly associated with RFS (*P* = 0.012, *P* = 0.034 and *P* = 0.003, respectively), although age, tumour size, deep stromal invasion and LVSI were not. As for OS, significant differences were detected in histology and PLN metastasis (*P* = 0.026 and *P* = 0.006, respectively). The M+ patients tended to have worse RFS (*P* = 0.064) and OS (*P* = 0.076) than the M- patients. The Kaplan–Meier curves of RFS for all patients with CC are shown in Fig. 2a. Regarding SCC, RFS was significantly shorter in the M+ patients than in the M− patients (*P* = 0.041) (Fig. 2b). However, no significant differences between M+ and M− were observed in the RFS of the non-SCC patients (Fig. 2c).

**Table 3:** Comparison of relapse-free survival (RFS) and overall survival (OS) of clinicopathologoical characteristics and mutation.

	N	3-Year RFS (%)	*P* value	3-Year OS (%)	*P* value
Age (years)			0.793		0.761
< 45	25	76.0		91.8	
≥ 45	30	79.5		88.2	
Histology			0.012		0.026
SCC	36	85.3		97.0	
Non-SCC	19	61.8		77.3	
Tumor size (mm)			0.771		0.808
< 40	18	77.4		88.5	
≥ 40	37	77.4		90.7	
Deep stromal invasion			0.27		0.322
≤ 1/2	29	80.4		93.4	
> 1/2	26	73.7		84.5	
Parametrial involvement			0.034		0.336
No	40	82.3		91.9	
Yes	15	62.9		83.6	
LVSI			0.284		0.160
No	14	85.1		100	
Yes	41	74.7		86.2	
Positive pelvic node			0.003		0.006
No	31	93.1		100	
Yes	24	57.7		75.7	
Mutation			0.064		0.076
Negative	20	90.0		100	
Positive	35	69.8		84.1

## Discussion

Our findings showed high rates of a potential oncogenic driver or drug targetable mutations in CC patients (Fig. 1). Validated mutations have been reported in 34–60% of CC patients using a cancer panel or whole exome sequence^12,13,17^. In the present study, 63.6% (n, 35/55) of the CC patients had more than one somatic mutation in either one or multiple genes (Fig. 1), and this rate was similar to those reported in previous studies^12,15^. We investigated the differences in mutation frequency between SCC and non-SCC patients. The frequency was higher in the non-SCC (73.7%, 14/19) patients than in the SCC (58.3%, 21/36) patients; however, the difference was not statistically significant (Table 1). Spaans et al*.* also reported that no significant differences in somatic mutation rates were observed between histological subtypes (36% in SCC, 38% in AC, and 28% in adenosquamous carcinoma)^17^.

Oncogenic mutations in *PIK3CA* lead to deregulation of the phosphatidylinositol 3-kinase-Akt signalling pathway, including tumour development, cell survival and cellular transformation, and are reportedly the most common genetic alterations in CC^12,15^. In the present study, the gene with the highest mutation rate was *PIK3CA* (34.5%) (Table 1). This prevalence is substantially similar in other investigations (23–33%)^9,13,18^. Although there was no significant difference, the *PIK3CA* mutation rate was higher in the SCC patients than in the non-SCC patients (41.7 and 21.1%, respectively) in the present study. Wright et al*.* also described a higher mutation rate in SCC tumours than in AC tumours; however, the difference was not statistically significant, which was most likely due to their study’s small sample size^13^. Furthermore, other studies have reported that *PIK3CA* mutations were more common in SCC than in non-SCC tumours^13,17^. *KRAS* is the most commonly mutated oncogene in human cancer, and its mutation is generally associated with resistance to treatment and poor survival^19^. In the present study, *KRAS* mutation was detected in 26.3% of the non-SCC patients, compared to none in the SCC patients (*P* = 0.003) (Table 1). A similar difference between AC and SCC in the uterine cervix was reported by others^13,17^. The *PTEN* gene is mutated in a wide variety of sporadic cancers at high frequency, and the protein encoded by this gene down-regulates intracellular phosphatidylinositol-3,4,5-trisphosphate levels, and functions as a tumour suppressor by negatively regulating the AKT/PKB signalling pathway^20^. In the present study, *PTEN* mutations were detected in 7.3% (4/55) of all CC tumours, but there was no difference between the SCC and non-SCC tumours (Table 1). The mutation genes in the current study include the *PIK3CA* (34.7%), *KRAS* (9.1%), and *PTEN* (7.3%); all are crucial members of cell cycle regulation. Importantly, tumours with these mutations can be amenable to targeted therapy in CC^21^. Somatic mutations in the *TP53* gene are well-known genetic alterations in malignant tumours^22^. The reported proportions of uterine cervical AC with mutated *TP53* have varied from 4% in North America to 19% in Asia^10^. In the present study, we observed that 9.1% of CC patients had mutations in the *TP53* gene (Table 1). The prevalence of *TP53* mutations was significantly higher in the non-SCC (21.1%) patients than in the SCC (2.8%) patients (Table 1). The difference in the prevalence of *TP53* mutation between the non-SCC and SCC patients was similar to that of a previous study by Tornesello *et al*^10^.

We investigated the associations between somatic mutation status and risk factors of CC. A relationship between M+ and PLN metastasis in early-stage CC after RH was observed (Table 2). To the best of our knowledge, such data have not been reported so far; we therefore believe that this is a new finding. As for other cancer types, concurrent driver mutations have been reported to be significantly correlated with lymph node metastasis in non-small cell lung cancer^7^. Muller et al. reported that the majority of breast cancer metastases have potentially actionable mutations^23^. If a biopsy sample can be analysed for somatic mutations before treatment, M+ may have clinically important information that is useful for predicting PLN metastasis in CC. No significant differences in mutation frequencies were observed in our tumour subgroups, according to other clinical characteristics (Table 2).

In the present study, M+ CC patients had a worse RFS than the M- CC patients (Fig. 2a). In particular, a significant difference was observed in the RFS of the CC patients with SCC (Fig. 2b). Our data also detected a significant relationship (*P* = 0.026) between M+ and PLN metastasis in the CC patients with SCC (data not shown). In a study by Spaans et al*.*, a positive mutation status correlated with worse RFS, and their survival data were similar to ours^17^. For patients with early-stage CC, lymph node metastasis has been proven to be a critical risk factor for survival^24–26^. In the current study, PLN metastasis was the worst clinicopathological prognostic factor as shown in Table 3, PLN metastasis may be a confounder in the relationship between mutation and survival. Our data suggest that driver somatic mutations are associated with PLN metastasis in early-stage CC after RH, and could be associated with a higher recurrence rate as a result.

As for the relationships between each somatic mutation and survival, *PIK3CA* mutations were associated with poor survival in CC patients^9,13^. In contrast, postoperative early-stage CC with *PIK3CA* mutations in Chinese patients was reported to be associated with a longer RFS and a lower likelihood of distant metastases in both univariate and multivariate analyses^18^. In the present sturdy, there was no significant association between *PIK3CA* mutations and RFS (Supplementary Fig. S1). The association between *PIK3CA* mutation status and prognosis is controversial. *KRAS* mutations have been linked to higher rates of tumour relapse in patients with non-SCC of the cervix^11^. *TP53* gene aberrations (deletion or mutations) have been reported to be associated with worse survival; CC patients with mutated *TP53* had the poorest prognosis^27^. Although *CTCNB1* mutations in CC patients are rare, a significant association between CC recurrence and cervical SCC has been reported^16^. We were unable to investigate the relationships between *KRAS, TP53, CTCNB1* mutations and RFS because there were too few M+ patients in the current study. Although each single gene mutation was not associated with survival, M+ tended to be associated with survival and was significantly associated with RFS in the SCC patients. Our data indicate that evaluations of mutations in early-stage CC should target multiple genes in order to identify risk factors and decide on the appropriate treatment.

Although many oncogenic gene mutations were analysed in the current study, there were some limitations. The main limitations were the retrospective design, the small sample size and short follow-up period that did not provide the proper evaluation of the clinical significance of somatic mutations in terms of survival and therapy outcomes. Making a determination as to whether mutation status can show relevant information for the management of individual patients with different clinicopathological types of CC requires prospective longitudinal studies. Another limitation of the current study is that our mutational analysis is incomplete, as it was limited to hot-spot variants (n = 2790) in a relatively small set of genes (n = 50). More comprehensive analysis, such as whole exome or genome sequencing, would be more informative to further understand the possible relationships between genomic alterations and clinicopathological factors or survival. Next, although persistent infection with high-risk human papilloma virus (HPV) is associated with significant risk of high-grade cervical intraepithelial neoplasia, and might play important roles in the development of CC, HPV genotyping was not routinely analysed for CC patient management, because such genotyping is not typically necessary for management of advanced CC. The E6 and E7 oncoproteins encoded by high-risk HPVs interfere with the function of p53 and Rb tumour suppressors, and leading to malignant transformation in CC^28^. Previously, p16 and Ki67 were reported to be a surrogate marker for the prediction of high-risk precursor or invasive CC lesions. Recently, it has been reported that PDL1 may be a useful biomarker to differentiate CIS (carcinoma in situ) from microinvasive cancer and, thus, anti-PDL1 therapy may inhibit the progression of CIS to the invasive stage^29^. The association between somatic mutation and these biomarkers related to HPV infection may include an important factor for the iniciation and/or development of CC. Finally, carcinogenesis is a multistep process characterized by accumulation of somatic mutations, which contribute to initiation, promotion, progression, and metastasis. Since lymph node metastasis is the final phase in carcinogenesis, the M+ detected in this study cannot be described as having a direct bearing on lymph node metastasis.

In summary, our data for the first time indicate an association between M+ and PLN metastasis in early-stage CC tumours after surgery. Furthermore, our M+ patients tended to have poor survival; especially, those with SCC had a significantly higher recurrence rate than the M- patients with SCC. These results may provide an important opportunity to predict lymph node metastasis and improve outcomes in patients with early-stage CC. In future, prospective controlled studies should be conducted to confirm these findings in larger populations of patients with long-term follow-up using whole exome or genome sequencing.

## Materials and methods

### Tissue specimens

A total of 55 surgical primary specimens were obtained from CC patients who had undergone RH including pelvic lymphadenectomy at Fukushima Medical University Hospital in Japan between June 2014 and December 2017. Pathological diagnosis for histologic cell type, tumour size, deep stromal invasion, parametrial invasion, LVSI, PLN metastasis and staging (FIGO 2008) was performed by experienced pathologists. All enrolled patients were pathologically diagnosed as having stage IB-IIB CC. Adjuvant therapy was determined according to the physician's treatment strategy; the treatment options included no further treatment, radiation alone, radiation therapy with chemotherapy, and chemotherapy alone. Patients who had undergone pre-operative treatment were excluded. Clinical and histological parameters were collected from medical records by the patients’ attending physicians, and were then coded and blinded from patient identification. The study design was approved by the ethics committee of Fukushima Medical University (No. 1953), and informed consent was obtained from all participants. All experiments were performed in accordance with the relevant guidelines and regulations.

### DNA extraction

Isolation and purification of genomic DNAs after extraction of RNAs from 55 frozen tumour samples were performed using ISOGEN reagent (Nippongene, Tokyo, Japan) according to the manufacturer's instructions. The concentration and quality of each DNA sample were assessed using NanoDrop One (ThermoFisher Scientific, Waltham, MA, USA).

### Somatic mutation detection

The Ion Ampliseq Cancer Hotspot Panel v2 on the Ion Torrent platform was performed to detect 2790 mutations in 50 oncogenes and tumour suppressor genes^16,30^. In brief, 10 ng of genomic DNAs extracted from 55 frozen tumour samples were used to construct barcoded DNA libraries utilizing the Ion Ampliseq Library Kit 2.0 (Thermo Fisher Scientific). The obtained libraries were optimized using Ion Library Equalizer Kit (Thermo Fisher Scientific), and then sequenced using an Ion Personal Genome Machine or Ion S5XL platform (Thermo Fisher Scientific). The sequencing reads were aligned to the reference genome build hg19, GRCh37, and converted into BAM files using Ion Torrent Suite software (Thermo Fisher Scientific). Sequence variants were called using Ion Reporter 5.0 (Thermo Fisher Scientific), according to the manufacturer’s instructions. The average sequencing depth reached at least 1500-fold per sample.

### Statistical analysis

The associations between validated mutations and histology, as well as those between somatic mutations and categorical variables, were evaluated using the chi-squared or Fisher’s exact test. RFS and OS were evaluated as clinical outcomes. Survival distributions were calculated by the Kaplan–Meier method, and statistical significance was determined using the log-rank test. Values of *P* < 0.05 were considered statistically significant. Statistical analysis of data was performed using SPSS version 25 software (SPSS, Inc., Chicago, IL, USA).

## Electronic supplementary material

Below is the link to the electronic supplementary material.

Supplementary Information
