# Supplementary material for: Clinical implication of oncogenic somatic mutations in early-stage cervical cancer with radical hysterectomy
Source: Sci Rep. 2020 Oct 30;10:18734. doi: 10.1038/s41598-020-72518-1 (PMC7599240; doi:10.1038/s41598-020-72518-1)
Supplement: Supplementary file 1 — Supplementary Information [file 41598_2020_72518_MOESM1_ESM.docx]

**Clinical implication of oncogenic somatic mutations in early-stage cervical cancer with radical hysterectomy**

Takafumi Watanabe^1^, Hideaki Nanamiya^2^, Manabu Kojima^1^, Shinji Nomura^1^, Shigenori Furukawa^1^, Shu Soeda^1^, Daisuke Tanaka^2^, Takao Isogai^2^, Jun-ichi Imai^2^, Shinya Watanabe^2^ and Keiya Fujimori^1^

^1^Department of Obstetrics and Gynecology, Fukushima Medical University School of Medicine, Fukushima, 960-1295, Japan

^2^Medical-industrial Translational Research Center, Fukushima Medical University School of Medicine, Fukushima, 960-1295, Japan

Correspondence to: Takafumi Watanabe, Department of Obstetrics and Gynecology, Fukushima Medical University School of Medicine, Fukushima 960-1295, Japan

E-mail: t-wata@fmu.ac.jp


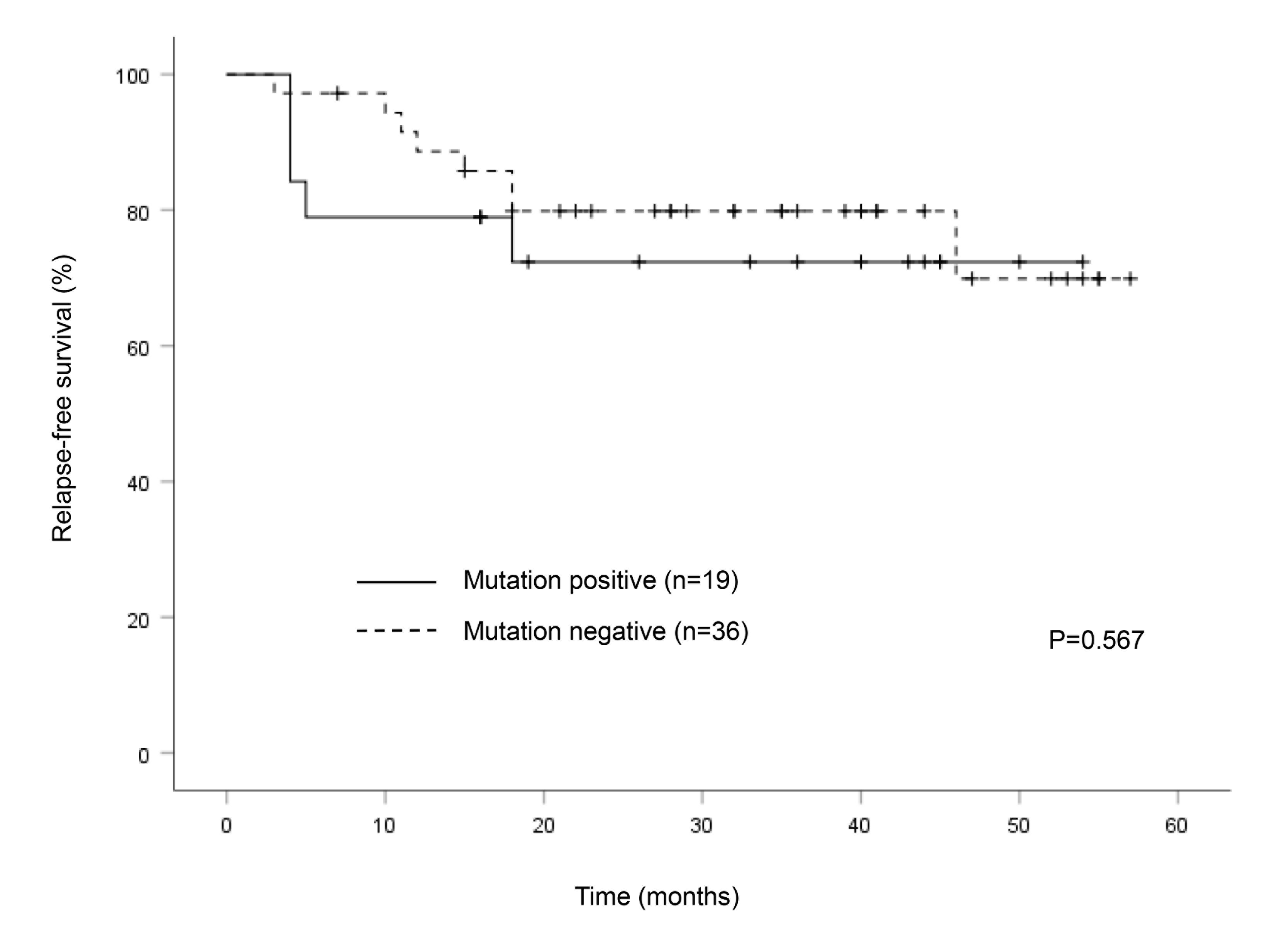


**Supplementary Fig. S1** Kaplan-Meier curves showing relapse-free survival according to *PIK3CA* mutation status in patients with cervical cancer.
